# Scientific Items

**Published:** 1883-05-20

**Authors:** 


### SCIENTIFIC ITEMS.

**Time by Telegraph.**—A company was incorporated at Albany recently for the manufacture, use, lease and sale of instruments for the indication of time by telegraph. The idea upon which the company is based is a good one. This company purposes to place central clocks in different parts of the city, which will be carefully regulated and frequently compared with some standard time-piece. Synchronized dial-plates connected with these clocks will be furnished to whoever desires them at a regular tariff. Several of these synchronized dial-plates are now in use in the city. There is one in Cooper Union, and two have recently been placed in a Broadway window of the Western Union Telegraph office, one indicating New York time and the other Chicago time. These latter are connected with the large clock in the office of James Hamblet, superintendent of the time telegraph department on the fourth floor of the building. The minute hand on these clocks springs forward one space on the first second of each minute. Thirty miles of wire, running into one hundred offices in New York, Brooklyn and the neighborhood, is connected with Mr. Hamblet's clock. A bell is struck in each office on every quarter hour, by which time-pieces may be regulated. Important enterprises in time telegraphy are on foot, and will shortly be made public.—*Mechanical News.*

**Iron Shot.**—There have been many experiments made to produce a good merchantable article of iron shot, and, according to a report coming from Iowa, the efforts in this direction are about to be of some avail. An exchange says that a company has been formed in that State for the manufacture of sporting shot from that material, and that the trials that have been made have proved them equal to the shot made from lead. No tower is said to be required, as the shot is made by the new process with less than three feet drop.—*Ibid.*

**Melting Steel.**—Jacob Reese, of Pittsburg, Pennsylvania, puts forth some remarkable claims in regard to an alleged new discovery in metallurgy. He says he is able to melt instantly a bar of cast-steel one inch in diameter—which cannot be fused in less than five minutes in the highest furnace heat attainable—simply by throwing against it a column of air having a velocity of 25,000 feet per minute.—*Ibid.*

**A Non-Conductor** of electricity has yet to be found, for all substances hitherto discovered are conductors of the force under certain known conditions; but those which offer a great resistance to it, serve the purpose of non-conductors in practice, although they may be all classed as good or bad conductors. The best conductor known at present is silver, the worst conductor is solid paraffine.—*Ibid.*

**An Instantaneous Light**.—Such in a word is the unique apparatus on exhibition at the rooms of the Portable Electric Light Co., 22 Water Street, Boston. It occupies the space of only five square inches and weighs but five pounds, and can be carried with ease. The light, or more properly lighter, requires no extra power, wires or connections, and is so constructed that any part can be replaced at small cost. The chemicals are placed in a glass retort; a carbon and zinc apparatus, with a spiral platinum attachment, is then adjusted so as to form a battery, and the light is ready. The pressure on a little knob produces an electric current by which the spiral of platinum is heated to incandescence. The Portable Electric Light Company was recently incorporated, with a capital of $100,000 under the laws of Massachusetts. The usefulness of the appartus and the low price ($5) will no doubt result in its general adoption. Some of the prominent business men of the State are indentified with this enterprise. In addition to its use as a lighter, the apparatus can also be used in connection with a burglar alarm and galvanic battery.—*Boston Transcript*, *Dec 30.*

Among the various uses to which electricity may be put there is one of a very practical nature, which promises to effect a great saving of property and life. It consists of an arrangement for the immenidate stoppage of a steam engine by merely pressing a button similar to those by which electric bells or fire alarms are sounded. This button may be placed at any distance from the engine upon which it acts; and Mr. Tate, the inventor, proposes that a number of such buttons should be dispersed throughout the factory or elsewhere where the appaiatus is in use. In factories accidents occur almost daily through the impossibility of stopping machinery on the instant. Such accident will readily be avoided by this method of instantaneously stopping the engine from any part of the building in which it works. The principle of the contrivance depends on the action of an electro-magnet upon the stop valve of the engine.—*Christian Advocate.*

It is well known that minute metallic particles are often collected in places remote from terrestrial sources of dust. Recent investigation shows that many of these particles must have undergone combusion, which evidently proves that they have come from the smoke of factories, from volcanic fires, or that they had a meteoric origin. It is found by chemical analysis that in addition to iron they contain nickle and cobalt, and neither of these two substances have ever been known to exist in similar particles form factory smoke or from volcanic dust. The evidence is therefore on the side of many who have maintained that the so-called meteoric dust realy comes to us from space.

The Pictorial World, published in London, England, has ordered a balloon which is to be provided with experienced æronauts, who will make atmospheric voyages, take balloon photographs, and report the results in the journal, with illustrations.

